# Physicochemical properties and shelf-life of low-fat pork sausages wrapped with active film manufactured by sodium alginate and cherry tomato powder

**DOI:** 10.5713/ajas.20.0132

**Published:** 2020-05-12

**Authors:** Zhuang Zhuang Qiu, Koo Bok Chin

**Affiliations:** 1 Department of Animal Science, Chonnam National university, Gwangju, 61186, Korea

**Keywords:** Low-fat Sausage, Active Film, Cherry Tomato, Physicochemical Properties

## Abstract

**Objective:**

This study was carried out to investigate physicochemical properties, and antioxidant and antimicrobial activities of low-fat sausages (LFSs) covered with sodium alginate (SA) film alone and with powder film (TSA-film) formed by cross-linking cherry tomato powder (CTP) and SA with calcium chloride (CaCl_2_).

**Methods:**

Sausages covered with the biodegradable film were assessed based on the measurement of pH, color (L*, a*, b*), proximate analysis, expressive moisture (EM), texture profile analysis, total plate counts (TPC), violet red bile, and 2-Thiobarbituric acid reactive substances (TBARS) during storage under refrigeration. LFSs wrapped with TSA-film were compared with those wrapped with SA-film and without film (control) during storage at 10°C for 35 days.

**Results:**

The LFSs covered with the mixed film had lower pH, lightness (L*), EM%, TBARS, and TPC, but lower yellowness (b*) and hardness values than those wrapped with TSA-film alone.

**Conclusion:**

Lipid oxidation and microbial growth was retarded in sausages covered with biodegradable films, especially multiple films as compared to single film, thereby resulting in extended shelf-life of the LFSs.

## INTRODUCTION

In recent years, developments in biodegradable polymer packaging films have been extensively studied. Environmental and food safety issues have resulted in increased interest in the exploitation of packaging materials obtained from natural resources to extend the shelf-life of foodstuffs. Moreover, traditionally used synthetic packaging materials had problems related to the food safety issues which necessitate the need for replacement of these with natural resources. The concerns of health-conscious consumers are emerging about food safety issues and therefore, current research is focused on the use of natural, active, edible, and biodegradable packaging materials for extension of shelf of low-fat sausages (LFSs) [1].

Alginate is a promising natural polymer that has been used widely for controlled release of bioactive compounds due to its peculiar characteristics, such as biocompatibility, biodegradability, and remarkable ability to form hydrogels [2]. Its matrix can be hydrated to form a gelatinous layer under relatively mild chemical conditions; also cross-linking of alginate matrix with multivalent cations, such as calcium, has been reported [3]. Alginate could be divided into two broad categories: homopolymeric (M-M and G-G) and heteropolymeric (MG or GM) substances [4]. Binding of Ca^2+^ to alginate is carried out by the three-step process involving mono-complexation, dimerization, and subsequent combination of dimers [5]. The diffusion of small molecules is facilitated by nonporous alginate gel (pore size ~ 5 nm) [6].

Cherry tomato has been one of the most widely con sumed food commodities. They are rich source of lycopene, polyphenols, carotenoids, minerals, vitamin C, and small quantities of other vitamins [7]. Among them, lycopene is the most abundant pigment which imparts red color to tomatoes [8]. Lycopene along with carotenoids provides provitamin and antioxidant properties; furthermore, rich nutritional profile and delicious taste have drawn attention towards this widely consumed fruit over the past years [9,10]. Tomato powder is produced by drying methods to reduce internal moisture to proper amount. They are an ideal candidate as additive to a variety of food products: soups, sauces, marinades, meat products, dips, cereal products, fruit purees, and snacks owing to their characteristic color, flavor, and water binding properties [11]. Nutrient degradation has been reported to occur during drying of fresh fruits and vegetables [12,13]. However, conversion of tomatoes into powder form has several advantages like shelf stability and wide applicability to different food products, mineral mixtures, supplements, and anti-aging products [11]. Fernández-Pan et al [14] evaluated the antimicrobial effect of edible coatings and demonstrated effectiveness in case of chicken breast with regard to control of microbial growth than other common preservatives. Edible films in combination with essential oils have been employed to achieve improvement in preservation and enhancement of shelf-life. In order to ensure safety and preservation of product quality during storage, controlled release of active ingredients is an important phenomenon in application of functional packaging. In current study, we added 1% cherry tomato powder (CTP) to sodium alginate (SA) and the effects of active film on physicochemical properties of LFSs, functional compounds, and the extension of shelf-life were assessed.

However, there exist only a few studies on the application of composite film for packaging of meat products. Thus, the objective of this study is to evaluate physicochemical properties and antioxidant activity of alginate films added with CTP as influenced by cross-linking agent (calcium chloride). The active film was found to be beneficial due to its potent antimicrobial and preservation effects in terms of extending the shelf life of foodstuffs.

## MATERIALS AND METHODS

### Cooked pork model sausages preparation

Pork hams (Landrace×Yorkshire, grade A) were purchased from the local market (Samho Co., Gwangju, Korea). After taking off the lean and excess connective tissue, the meat was ground and mixed with non-meat ingredients (1.3% salt, 0.4% sodium tripolyphosphate, 0.25% cured blend, 0.05% sodium erythorbate, and 1% soy protein isolate). The meat batter was stuffed into polyvinylidene dichloride film and cooked in a water bath at 75°C for 30 min. After chilling, LFSs were coated with films and stored at 10°C under refrigeration for 35 days.

### Film preparation

The SA was purchased from the Tureban company (Goyang, Korea). The CTP was purchased from seed fruit and vegetable products company (Dunhuang, Gansu, China). A film-forming solution including SA (2%) was dissolved in calcium chloride solution (0.01 g/100 mL) with glycerol (same amount as SA) and dried in a dry-oven at 50°C for 24 h. Cross-linking was completed by pouring 50 mL of calcium chloride into film for 30 s followed by drying at room temperature for 6 h. Except that, 1% CTP was additionally added to prepare for a tomato SA film.

### pH, color value, and microbial count

The pH of samples was determined randomly with a pH meter (Mettle-Toledo, Schwarzenbach, Switzerland). The color values consisting of lightness (Commission Internationale de l’Eclairage [CIE] L*), redness (CIE a*), and yellowness (CIE b*) of samples were measured by the color reader (Model CR-10. Minolta, Tokyo, Japan). For the microbial count, 10 g ground sample was mixed with 90 mL saline water (0.9%) using a Stomacher Lab Blender and serial dilutions were prepared. Then, about 0.1 mL of diluted sample was dispersed onto the surface of violet red bile (VRB) and total plate count (TPC) agar (Bingol and Bostan [15]). Subsequently, VRB and TPC agars were incubated at 37°C for 24 h.

### Proximate composition

Moisture, crude fat content, and fat content were assessed at 0, 3, 7 14, 21, 28, and 35 days by the method of AOAC [16]. Crude fat content was determined by the Soxhlet extraction method and crude protein analysis was carried out by following Kjeldahl technique.

### Texture profile analysis

Universal Testing Machine (Model 3344, Canton, MA, USA) was used to perform texture profile analysis (TPA) according to the method described by Bourne [17]. Sausage samples (1.30 cm length and 1.30 cm diameter) were compressed with a 500-N load cell at operational speed of 300 mm/min. TPA values were expressed in terms of hardness (gf), springiness (cm), gumminess, chewiness, and cohesiveness of LFSs.

### Expressible moisture

Approximately 1.5 g of sample was wrapped in 3/4 filter paper (one piece of filter paper was separated into 4 pieces uniformly). Then, the sample was centrifuged (3,000 rpm) for 15 min (VS-5000N, Vision Scientific Co. Ltd., Bucheon, Korea). Weight of both filter paper and samples was measured again. The expressible moisture (EM) content of samples was calculated as follows:

EM (%)=ΔT×100/A

ΔT was the Thimble weight difference before and after cen trifugation. A was the initial weight of the sample.

### Thiobarbituric acid reactive substances

The oxidative rancidity was evaluated based on the thiobarbituric acid reactive substances (TBARS) value [18]. Each sample was mixed with 2.5% trichloroacetic acid (3 mL) and 1% 2-thiobarbituric acid (17 mL) in a cap tube. Then, tubes were put into 90°C boiling water bath for 30 min. Next, supernatant of each solution was mixed with 5 mL chloroform and centrifuged at 2,000 rpm for 5 min (VS-5000N, Vision Scientific Co. Ltd., Korea). Subsequently, approximately 3 mL petroleum was added to each supernatant and further centrifuged. Finally, clear solutions were analyzed by spectrophotometer (UV-1601, Shimadzu, Kyoto, Japan) at a wavelength of 532 nm.

### Statistical analysis

The data on the effects of three different treatments during 35 days of storage at 10°C on physicochemical properties and antioxidant activities of LFSs were collected and subjected to two-way analysis of variance with treatments and storage time as factors using SPSS 21.0 program for windows. If the interaction between two factors were significant, then the data were separated out by treatment and storage time, which was analyzed at the significant level of 0.05, respectively.

## RESULTS AND DISCUSSION

### pH and color

pH and color values of LFSs are shown in Table 1. During storage, no changes in pH values of pooled control and treatments were observed during storage time, since the treated sausages and control didn’t detect microbial counts until 28 days of storage (< 2^e^ Log colony-forming unit [CFU]/g). LFSs wrapped with 2% SA film were same pH as LFSs without film (control [CTL]), but LFSs wrapped with 2% SA and 1% CTP film (TSA film) were lower pH than those of control, which might be due to the addition of tomato powder. Although the pH of sausage decreased with the wrapping with TSA-film statistically, the differences in pH was not enough to affect the sausage quality of sausage since the difference in pH was minimal. However, Deda et al [19] reported that the pH reduction of frankfurter containing tomato paste was a result of an increase in lactic bacteria during storage. Surface color is the most important factors affecting consumer acceptance, purchasing decisions, and satisfaction. Sausage color values were primarily related to the film. LFSs with TSA film had lower lightness (L*) value, but higher yellowness (b*) value than CTL. Redness (a*) values of all treatments exhibited no change regardless of wrapping. Based on the results, it was apparent that improvement in red and yellow color was due to the wrapped films, which led to increased preference of consumers for the products. Yuan et al [20] reported that chitosan films with added pomegranate peel extracts reduced the lightness and increased the redness. The increment of yellowness was affected by the lycopene in tomato powder, which imparted a peculiar red color [21]. During storage time, the redness and yellowness trended to increase, but lightness didn’t change. Candogan [21] did the similar study on beef patties added with different levels of tomato paste and he found that during storage, lightness and redness of patties added with tomato paste decreased but yellowness increased. He also reported that changes of color values were due to antioxidant activities of lycopene and the pigments present inside tomato paste.

### Proximate analysis

Crude fat, crude protein, and moisture of CTL, SA, and TSA were 2.05, 2.23, and 2.26; 16.30, 17.04, and 17.45; 80.3, 78.8, and 78.6, respectively (Table 2). During the storage period, moisture and crude fat contents (%) exhibited no differences, except for a gradual decrease in crude protein level. Although moisture and crude fat contents (%) of all treatments were almost similar, regardless of wrapped films, protein content (%) of LFSs wrapped with TSA film was higher than CTL and similar to LFSs wrapped with SA film; However, no differences in protein content (%) was observed between CTL and LFSs wrapped with SA film (p>0.05). Elbakheet et al [22] reported that significant differences in fat content, ash content, and fiber content (%) were not observed among different samples during storage. Also, protein content, fat content, ash content, and crude fiber content exhibited an increase with increased addition of replacement of wheat germ flour. Increased protein content (%) in LFSs wrapped with film was observed, especially in LFSs wrapped with TSA film. However, moisture or fat content (%) was not different among the different treatments. Based on proximate analyses, it can be concluded that LFSs wrapped with film affected the proximate analyses due to water absorption from sausages by the film thereby, resulting in increased protein content. On the other hand, no changes in fat (%) were observed, since the sausages had very low-fat content (%).

### Texture profile analyses

**Figure 1:**
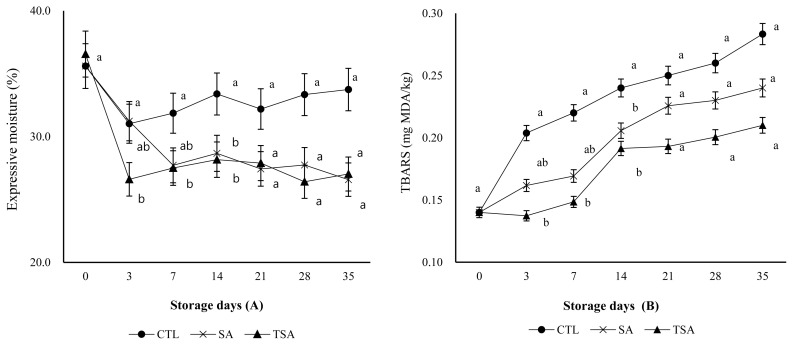
Expressive moisture (A) and thiobarbituric acid reactive substances (B) of low-fat pork saussages and those wrapped with SA and TSA films. EM, expressive moisture; TBARS, thiobarbituric acid reactive substances; CTL, low fat sausages without film; SA, low fat sausages wrapped with 2% sodium alginate film; TSA, low fat sausages wrapped with 2% sodium alginate and 1% cherry tomato powder film. ^a,b^ Mean with different superscript on the same day indicate significant differences at p<0.05.

The results of texture profile analyses of LFSs during storage are shown in Table 3. LFSs wrapped with the film were harder than CTL, but those wrapped with TSA exhibited texture similar to the ones wrapped with SA. Ruiz-Ramírez et al [23] reported that hardness of textural was affected by pH and water content. In this study, the increases in hardness (gf) could be explained based on lower expressive moisture (Figure 1A) and pH (Table 1). Additionally, it has been widely recognized that water absorption by film affects textural hardness (gf). The water content in LFSs was absorbed by the film thereby resulting in increased hardness (gf). However, springiness (mm), gumminess (kg/mm), chewiness (kg/mm), and cohesiveness (ratio) of all LFSs were not different among the treatments. Sánchez-Ortega et al [24] found that films could extend the shelf life because physicochemical properties (such as texture, moisture) of meat or meat products changed due to the gas barrier properties of film. They also reported that application of film also changed the sensory evaluation due to the changed physicochemical properties of the sausages. Our study was agreement with theirs and film or coating technique should increase the shelf life or quality and safety of meat or meat products without changes in sensory characteristics. Although film absorbed water from sausages, it didn’t much affect the sausage quality due to the mechanical properties (moisture content, solubility in water, water vapor permeability) of films [24,25]. Alginate coating could reduce microbial counts and form acceptable flavor, tenderness and appearance [26]. Thus, application of films to LFSs might change the textural hardness and may also affect the sensory characteristics.

### Microbial counts

As shown in Table 4, TPCs of treatment covered by TSA film were detected from 28 days of storage days and were found to be lower than CTL and LFSs covered by SA film. The lower microbial count in sausages wrapped with the TSA-film might be attributed to lower pH value, which resulted in reduced microbial growth. Decreased pH inhibited the anaerobic growth of enterobacteria and prevented meat spoilage as supported by previous studies. Grau [27] reported that lower pH value of muscle could support more undissociated lactate, which is an effective inhibitor. Vergara and Gallego [28] studied different compositions of gas present in the package during the 17 days of storage of lamb meat, and reported that pH values near 5.8 among the lower pH values favored bacterial growth and meat spoilage, which the relationship between bacteria and pH value was clearly reported. Another study also reported that modified atmosphere packaging with aloe gel or inclusion of thymol or eugenol enhanced the content of bioactive compounds including phenolics and lycopene, which in turn reduced the incidence of microbial spoilage [29]. These results indicated that LFSs wrapped with TSA film exhibited antimicrobial effect. It is proposed that TSA film can be used for enhancing the shelf-life of meat products.

### Expressible moisture (%) and thiobarbituric acid reactive substances

As shown in Figure 1A, EM of LFSs wrapped with TSA film was lowest among the treatments at day 3 and lower than CTL before day 14. Since EM is related to water holding capacity and water activity, and it could be affected by the barrier properties of film such as water absorption [30]. Figure 1B shows the result of TBARS values as affected by LFSs wrapped with different films. The TBARS values of all samples gradually increased during storage and those of LFSs wrapped with TSA film was lower than the CTL (p<0.05) before day 14. However, no differences in TBARS between LFSs wrapped with SA film and CTL were observed except for those on day 14 (p>0.05). The TBARS value exhibited a gradual increasing tendency and below rancid value (1.0) during or at the end of storage. Thus, LFSs wrapped with TSA film might have antioxidant activity as compared to other treatments. Similar results were accounted for by Siripatrawan and Noipha [31], who reported that pork sausages with green tea film extended shelf-life. During storage, higher TBA was observed in control samples rather than LFSs wrapped with chitosan film and chitosan green tea film due to antioxidant activity of chitosan as well as its lower oxygen permeability characteristic [31,32]. These results suggested that lipid oxidation of sausage could be affected by bioactive film and antioxidant grape tomato powder. In this study, antioxidant activity of LFSs wrapped with TSA film showed better results than CTL. Also, it is proposed that TSA film could be used to enhance the antioxidant activities of meat products.

## CONCLUSION

Sausages wrapped with TSA films increased hardness, but decreased protein contents (%) and pH values. In addition, reduced lipid oxidation, inhibited microbial growth, and improved water holding capacity were observed in the sausages with the wrapping of TSA, resulting in the extension of shelf-life of low-fat meat products during storage. Consequently, TSA films could be applied to meat products to extend the shelf-life in meat industries.

## Figures and Tables

**Table 1:** pH and color of low-fat pork sausages wrapped with SA and TSA films

Parameters	pH	CIE L*	CIE a*	CIE b*
Treatments1)				
CTL	6.06^A^	73.71^A^	10.70^A^	5.91^B^
SA	6.00^AB^	73.2^AB^	10.94^A^	6.10^B^
TSA	5.95^B^	72.79^B^	10.94^A^	7.15^A^
Days				
0	6.08^a^	73.80^a^	10.20^c^	5.70^c^
3	5.99^a^	73.38^a^	10.40^bc^	6.28abc
7	5.99^a^	72.98^a^	10.91abc	6.10^bc^
14	6.01^a^	72.68^a^	10.98abc	6.9^a^
21	6.01^a^	73.18^a^	11.20^ab^	6.2^bc^
28	5.97^a^	73.50^a^	10.92abc	6.42^ab^
35	5.97^a^	73.22^a^	11.40^a^	6.53^ab^

CIE L*, lightness; CIE a*, redness; CIE b*, yellowness.

1) CTL, low fat sausages without film; SA, low fat sausages wrapped with 2% sodium alginate film; TSA, low fat sausages wrapped with 2% sodium alginate and 1% cherry tomato powder film.

a–c Mean with a different superscript in the same treatment were different p<0.05.

A,B Mean with a different superscript in the same storage days were different p<0.05.

**Table 2:** Proximate composition low-fat pork sausages wrapped with film of SA and TSA

Items	Proximate analysis (%)		

	Moisture	Crude fat	Crude protein
Treatments1)			
CTL	80.3^A^	2.05^A^	16.30^B^
SA	78.8^A^	2.23^A^	17.04^AB^
TSA	78.6^A^	2.26^A^	17.45^A^
Days			
0	80.4^a^	2.20^a^	17.99^a^
3	80.1^a^	2.03^a^	17.48^ab^
7	79.2^a^	2.05^a^	17.30abc
14	79.5^a^	2.40^a^	16.96abc
21	77.9^a^	1.99^a^	16.45^bc^
28	78.9^a^	2.22^a^	16.35^bc^
35	78.8^a^	2.38^a^	15.99^c^

1) CTL, low fat sausages without film; SA, low fat sausages wrapped with 2% sodium alginate film; TSA, low fat sausages wrapped with 2% sodium alginate and 1% cherry tomato powder film.

a–c Mean with a different superscript in the same treatment were different p<0.05.

A,B Mean with a different superscript in the same storage days were different p<0.05.

**Table 3:** Texture profile analyses of low-fat pork sausages wrapped with SA and TSA films

Items	Parameters				

	Hardness (gf)	Springiness (mm)	Gumminess (kg/mm)	Chewiness (kg/mm)	Cohesiveness (ratio)
Treatments1)					
CTL	2,852^B^	6.36^A^	24.8^A^	163^A^	8.21^A^
SA	3,299^A^	6.42^A^	28.1^A^	178^A^	8.49^A^
TSA	3,318^A^	6.23^A^	27.2^A^	168^A^	8.41^A^
Days					
0	2,447^c^	6.05^a^	21.8^b^	120^c^	8.75^a^
3	2,971^bc^	6.27^a^	21.9^b^	129^bc^	7.89^a^
7	3,173^ab^	6.26^a^	26.2^ab^	163abc	8.37^a^
14	2,977^bc^	6.67^a^	27.4^ab^	164^ab^	8.57^a^
21	3,585^a^	6.22^a^	32.1^a^	210^a^	8.72^a^
28	3,529^ab^	6.45^a^	30.6^a^	213^a^	8.24^a^
35	3,414^ab^	6.48^a^	27.1^ab^	178^ab^	8.03^a^

1) CTL, low fat sausages without film; SA, low fat sausages wrapped with 2% sodium alginate film; TSA, low fat sausages wrapped with 2% sodium alginate and 1% cherry tomato powder film.

a–c Mean with a different superscript in the same treatment were different p<0.05.

A,B Mean with a different superscript in the same storage days were different p<0.05.

**Table 4:** Microbial counts of low-fat pork sausages wrapped with SA and TSA films

Items	Parameters (Log CFU/g)	

	TPC	VRB
Treatments1)		
CTL	4.59^A^	<2^A^
SA	4.23^AB^	<2^A^
TSA	3.65^B^	<2^A^
Days		
0	<2^b^	<2^a^
3	<2^b^	<2^a^
7	<2^b^	<2^a^
14	<2^b^	<2^a^
21	<2^b^	<2^a^
28	4.04^ab^	<2^a^
35	4.28^a^	<2^a^

CFU, colony-forming unit; TPC, total plate counts; VRB, violet red bile.

1) CTL, low fat sausages without film; SA, low fat sausages wrapped with 2% sodium alginate film; TSA, low fat sausages wrapped with 2% sodium alginate and 1% cherry tomato powder film.

a,b Mean with a different superscript in the same treatment were different p<0.05.

A,B Mean with a different superscript in the same storage days were different p<0.05.
